# Unmasking early microglial remodeling in an Alzheimer’s disease mouse model

**DOI:** 10.3389/fncel.2025.1720382

**Published:** 2026-01-02

**Authors:** Priyanka Saminathan, Sara McArdle, Maija Corey, Namratha Nadig, Camille Fang, Alicia Gibbons, Mahati Rayadurgam, Sonia Sharma

**Affiliations:** 1 La Jolla Institute for Immunology, La Jolla, CA, United States; 2 Department of Medicine, University of California San Diego, La Jolla, CA, United States; 3 Laboratory for Inflammatory Immune Metabolism, Center for Integrative Medical Sciences, RIKEN, Yokohama, Japan

**Keywords:** microglia morphometry, image segmentation, QuPath, neuroinflammation, Alzheimer’s disease, Iba1 and CD68 quantification, morphological remodeling, hippocampus

## Abstract

Early neuroimmune remodeling is a critical yet understudied component of Alzheimer’s disease (AD) pathogenesis. To investigate microglial contributions to AD development prior to overt plaque deposition, we developed an open-source morphometric pipeline to systematically quantify hippocampal microglial structure and activation states in pre-plaque 5xFAD mice. Across ∼11,000 cells, we extracted multidimensional parameters including area, circularity, convex hull, branch points, nearest-neighbor distance, and nuclear features, alongside Iba1 and CD68 intensity measurements. While no significant overt gliosis was observed at this early stage, microglia from 5xFAD mice exhibited subtle trends toward increased structural complexity compared to wild-type controls. Importantly, significant sex-specific differences were detected within the CA1 subregion: male 5xFAD microglia displayed hyper-ramified morphologies consistent with enhanced surveillance states, whereas female microglia demonstrated greater density and a more reactive phenotype. Correlation analyses revealed a conserved association between microglial complexity and Iba1/CD68 expression, independent of sex or genotype, underscoring a fundamental link between cytoskeletal remodeling and phagolysosomal activity. These findings highlight the capacity of morphometric profiling to sensitively detect early, region-specific, and sex-dependent shifts in microglial phenotype before amyloid deposition. By integrating quantitative morphology with canonical molecular markers, this framework provides a robust and unbiased approach for characterizing microglial activation trajectories. Such early readouts may inform biomarker discovery and therapeutic strategies aimed at modulating microglial responses to delay or prevent AD progression.

## 1 Introduction

Neuroinflammation is a core dimension of Alzheimer’s disease (AD) pathobiology, (Asai et al., 2015; Falkon et al., 2025; Guillot-Sestier et al., 2021) with microglia, the brain’s resident macrophages, poised at the interface of risk, injury detection, and circuit remodeling. Genetic studies first implicated microglia in Alzheimer’s disease, as many AD risk variants map to microglial pathways (Wickstead, 2023). Notably, rare loss-of-function mutations in TREM2 markedly increase susceptibility and hasten disease progression, alongside additional variants affecting immune regulation and lipid-handling genes that converge on microglial function. (e.g., CD33, CR1, ABCA7) (Carling et al., 2024; Heneka et al., 2025; Tsui et al., 2025). These data argue that microglial signaling pathways can shift the disease trajectory rather than simply report downstream damage (Bellenguez et al., 2022; Guerreiro et al., 2013; Hollingworth et al., 2011; Jonsson et al., 2013; Jun et al., 2010; Naj et al., 2011). Functionally, early microglial responses shape the sequence from amyloid-β (Aβ) stress to synaptic failure and tauopathy. In mouse models, complement components such as C1q and C3 accumulate at synapses before overt plaques (Hong et al., 2016; Shahidehpour et al., 2025; Wu et al., 2019). Microglial complement signaling then drives early synapse elimination and memory deficits, while blockade of complement protects synapses and neurocognitive behavior (Hong et al., 2016; Shi et al., 2017). These studies situate microglia as proximal orchestrators of the earliest circuit changes that better correlate with cognition status than bulk amyloid burden. Microglia can both propagate pathology through inflammatory signaling and exosome-mediated spread of misfolded proteins (Asai et al., 2015; Falkon et al., 2025; Guillot-Sestier et al., 2021) and simultaneously contain disease progression through plaque compaction and clearance (Keren-Shaul et al., 2017; Spangenberg et al., 2019). These findings underscore the nuanced and context dependent nature of microglial interactions with pathogenic proteins. Consequently, modulating microglial activation and function rather than removing them from the equation is emerging as a more promising strategy to restore homeostasis in the brain.

At the cellular level, microglial state transitions are accompanied by stereotyped structural remodeling that encodes functional roles in surveillance, synapse interaction, and phagocytosis. During homeostasis, microglia display fine, highly ramified arbors with small somata and dynamic process motility; stressors and disease cues drive shifts through hyper-ramified, de-ramified/bushy, and amoeboid phenotypes (Green and Rowe, 2024; Madry et al., 2018; Nimmerjahn et al., 2005). At early, preplaque stages in AD, *in vivo* imaging shows microglia adopting these hyper-ramified, primed morphologies with heightened process motility and clustering around emerging Aβ seeds (Gyoneva et al., 2016; Hou et al., 2022; Jung et al., 2015; Meyer-Luehmann et al., 2008). Microglia accelerate rapid plaque compaction and form barrier-like assemblies that limit amyloid spread and neuritic dystrophy, suggesting a context dependent balance between containment and collateral damage (Casali et al., 2020; Condello et al., 2015; Hou et al., 2022; Jung et al., 2015). Understanding when and where this balance tips remains a central challenge for early-stage intervention in AD. Translationally, the field requires biomarkers and analytic frameworks that detect subtle, pre-plaque immune remodeling with spatial precision, quantify cell-state heterogeneity, and link these readouts to synaptic health and future lesion emergence.

High-throughput imaging pipelines for microglial morphology, such as the Convolutional Neural Network (CNN)-based classifiers developed in ischemia, demonstrate scalable, unbiased labeling and correctly capture hallmark activation transition (Leyh et al., 2021). However, fixed morphotype categories and non-AD validation can obscure the subtle, spatially patterned, and sex-dependent remodeling that defines early AD (Gyoneva et al., 2016; Hou et al., 2022). Accordingly, resolving early AD-relevant shifts requires continuous, feature-level morphometrics coupled to Iba1/CD68 and spatial statistics.

A second, underexplored dimension of microglial modulation is biological sex. Microglia exhibit sex-dependent developmental trajectories and transcriptomes even at baseline; males and females differ in microglial density, morphology, and immune reactivity across brain regions (Bobotis et al., 2023; Guneykaya et al., 2018; Hanamsagar et al., 2017; Kang et al., 2024). In parallel, APOE-ε4 confers a greater AD risk and stronger association with tauopathy in women, suggesting interactions between sex, lipid biology, and innate immune tone that could bias disease initiation and spread (Altmann et al., 2014; Buckley et al., 2019; Haney et al., 2024; Lee et al., 2023). Notably, female 5xFAD mice develop plaques earlier and show more pronounced gliosis, underscoring that sex differences are evident even in experimental models (Poon et al., 2023; Rodriguez et al., 2025; Sil et al., 2022). These observations motivate sex-aware analyses of early microglial remodeling in AD models and human cohorts.

Building on this framework, we focus on early microglial remodeling as a predictor and potential driver of downstream AD pathology using the 5xFAD mouse model. 5xFAD mice overexpress human APP (Swedish, Florida, London) and PSEN1 (M146L, L286V), and begin depositing extracellular Aβ by 2 months, with robust early microgliosis (Oakley et al., 2006). Despite lacking tau pathology and progressing faster than human disease, their aggressive amyloidosis and early microglial activation make them a useful system for probing pre-plaque and early-stage remodeling (Oakley et al., 2006). We hypothesize that in pre-plaque stages, microglia undergo region-specific and sex-modulated structural transitions, characterized by hyper-ramification in vulnerable circuits and local changes in cell density and spacing. We believe these findings foreshadow complement-tagged synapse loss and later Aβ pathology. To test this, we deploy a quantitative, open, and reproducible immunofluorescence image analysis pipeline optimized for the study of microglia at ages preceding dense plaque deposition. Our image-analysis workflow combines semi-automated segmentation with per cell morphometrics which include: cell and soma area/circularity, convex hull area/solidity, total process length, branching index, critical radius and value (Sholl analysis), Schoenen Ramification Index (SRI), cellular Iba1 and CD68 intensities, and population-level spatial statistics that include microglial density and nearest-neighbor distance (NND). Methodologically, this study leverages validated tools to enhance sensitivity to changes that are typically lost in 2D sections. Automated or semi-automated platforms reduce observer bias and scale to thousands of cells, while recent benchmarking underscores the discriminative value of convex hull and skeleton based features and encourages open, machine-learning-ready data standards. These advances, coupled with region-resolved sampling, allow us to map the topography of microglial remodeling across vulnerable circuits with statistical power.

## 2 Materials and methods

### 2.1 Animal handling

Transgenic 5xFAD B6SJL mice [006554 /MMRRC 034840/ B6SJL-Tg(APPSwFILon,PSEN1*M146L*L286V)6] were purchased from Jackson Laboratories. Age- and sex-matched non-transgenic littermates on the same B6SJL genetic background were used as controls, following the vendor’s recommended control strategy for 5xFAD mice (JAX stock #006554 / MMRRC #034840). Mouse experiments were performed with 6–8 week old mice of both sexes housed in specific-pathogen-free conditions at the La Jolla Institute for Immunology. All procedures involving mice were performed according to the Institutional Animal Care and Use Committee (IACUC) at La Jolla Institute for Immunology.

### 2.2 Histology

Mice were transcardially perfused with 10 ml PBS and 10 ml 4% PFA, and brains were removed from the skull and fixed for additional 48h. Following washing with PBS, brains were cryoprotected in 30% sucrose solution in PBS and frozen in OCT compound. Coronal sections were cut at 35 μm and stored in cryoprotectant (30% PEG300, 30% glycerol, 20% 0.1 M phosphate buffer, and 20% ddH2O) at −20C until staining. Sections were washed with PBS (210 min), and incubated with 10% normal donkey serum with 0.5% Tween 20 in PBS for 2h at RT. This and subsequent incubations were carried out with a laboratory rocker. Sections were transferred to a 2 ml tube for incubation with the primary antibody cocktail (rat anti-CD68, clone FA-11, 2 μg/ml, Biolegend cat #137001, and rabbit anti-Iba1, clone E404W, 0.2 μg/ml, Cell Signaling Technologies cat #17198S) overnight at 4C. Sections were washed with PBS with 0.1% Tween 20 (PBS-T, 3×5 min) using transwell membrane inserts to facilitate transfer of sections to new wells of a multiwell plate. Samples were stained with goat anti-rat IgG Alexa Fluor 568, and goat anti-rabbit IgG Alexa Fluor 647 (both at 4 μg/ml, ThermoFisher Scientific cat #A11077, and A32733, respectively) cross-adsorbed secondary antibodies for 2h at RT. Samples were washed with PBS-T (2×5 min), PBS (1×5 min), stained with Hoechst 33258 (10 μg/ml, 10 min), washed with PBS (3×5 min), and embedded in Prolong Gold using #1.5 coverslip. Images were captured on a ZEISS Axioscan 7 slides scanner with a 20× 0.8NA objective, using 385, 567, 630 nm LED illumination modules, a multiband filter cube, and a 712 M camera, resulting in Z-stacks with a 0.172 × 0.172 × 0.490 μm voxel size.

### 2.3 Image pre-processing

To reduce noise, improve spatial resolution, and reduce the effect of out-of-focus light, Z-stacks were deconvolved using the Huygen’s Essential software suite (v23.10, Scientific Volume Imaging). First, individual tiles of each channel were deconvolved with the quick maximum likelihood estimate algorithm, using a brick mode of 1. A maximum intensity Z-projection was calculated for each tile and saved as an .ome.tiff. The 2D projections were stitched together using a single slice from the original .czi image as a starting template. The final file was saved as a 32-bit, 3 channel .ome.tiff image.

### 2.4 Microglia segmentation

All image analysis was performed in QuPath v0.5.1 (Bankhead et al., 2017). The hippocampus region was manually annotated, excluding the granule cell nuclei layer in the dentate gyrus. The CA1 subregion was also outlined. Within the full hippocampus region, microglia segmentation proceeded in 2 steps. First, a pixel classifier was trained on the Iba1 channel to segment microglia processes as precisely as possible and detection objects were created (minimum size threshold = 0.2 μm). Separately, nuclei were segmented with Cellpose (Stringer et al., 2021) using the “nuclei” model on the Hoechst channel through the QuPath Cellpose extension. Microglial nuclei were identified as those that were at least 30% Iba1 positive by area, as measured with the Iba1 pixel classifier; all other nuclei were deleted.

A custom script merged the separate Iba1 and nucleus detections into microglia (see Github)^1^. Briefly, we collected all detections that were within 100 μm of a microglia nucleus. Nearby Iba1 detections were merged using binary dilation and contraction to fill in small gaps that may have arisen from incomplete labeling of thin dendrites. The area that was contiguous with the nucleus was segmented, labeled as a microglia, and expanded to cover the entire nucleus. Looping over all Iba1^+^ nuclei resulted in a set of overlapping microglia objects; any remaining Iba1^+^ objects that were not associated with a nucleus were deleted. Overlapping microglia were separated with watershed separation using the nuclei centroids as initial markers. Each nucleus and its associated microglia outline were assigned matching names. Finally, to reduce artifacts, cells were removed if the nucleus was touching the hippocampal boundary or if the nuclear area or total cell area was too small (<5 μm^2^ or <50 μm^2^, respectively), suggesting it was a cell fragment.

### 2.5 Microglia metrics

Built-in QuPath functions were used to measure the area, perimeter, major and minor Feret diameters, and convex hull area and perimeter of each cell and nucleus, from which we calculated circularity, solidity, convexity, and aspect ratio [see (Leyh et al., 2021) for definitions and Supplementary Table 1 for more information]. The area of each cell that was positive for CD68 (pixel intensity >1000 AU) and the average Iba1 fluorescence intensity were calculated. More complex cell features were measured in Fiji (Schindelin et al., 2012). Each cell was exported from QuPath as a binary image with the nucleus saved as an overlay. In Fiji, the soma region (nucleus expanded by 3 pixels) was deleted, the remaining processes were skeletonized, and the skeleton was analyzed (Arganda-Carreras et al., 2010) for length and branch points. Additionally, the Simple Neurite Tracer plugin (Arshadi et al., 2021) was used to calculate Sholl features (Ferreira et al., 2014) of the microglia shape, using the nucleus centroid as a starting point and measuring concentric circles every 0.25 μm from the edge of the nucleus out to 50 μm. All metrics were calculated based on the sampled data and Schoenen Ramification Index was defined as the critical value divided by the number of branches. Branch thickness was calculated as the cell cytoplasm area (cell area minus nuclear area), divided by the total branch length and the nuclear offset was calculated as the distance between the cell centroid and nucleus centroid.

Spatial relationships between cells were calculated in QuPath v0.5.1 using Delaunay triangulation. The mean neighbor distances were calculated for the cell centroids, using a maximum distance of 250 μm. Separately, to identify clumped cells, Delaunay triangulation was calculated for the nucleus objects themselves, with a maximum distance of 25 μm. Reactive microglia were identified as those with CD68 expression >= 35% area, a Branching Index >= 1, and a nucleus <25 um from at least one other nucleus.

### 2.6 Statistical Analysis

The shape, intensity, and spatial distribution metrics for all cells were exported as .csv files, which were loaded into Matlab (Mathworks) for further processing. In most cases, each hippocampus (or CA1 region) was summarized as a median of all cells in that region. The statistical significance of group comparisons were calculated in Prism v10 (GraphPad) using a 2-way ANOVA of the slide medians, with uncorrected Fisher’s LSD *post-hoc* test with single pooled variance.

To look at the relationships between parameters, the dataset of 10490 microglia were divided into deciles of 1049 cells based on area, circularity, or branching index. The cells in each decile group were split by genotype and then the average CD68% or Iba1 intensity of the cells in each group were calculated. To evaluate the profile of the reactive microglia, the 50 detected reactive cells were compared to the homeostatic cells from the CA1 region of the 3 matching female 5xFAD mice that had a significant number of reactive cells. Outliers were removed using the ROUT test (with Q = 1%) and then shape metrics were compared with an unpaired, two-tailed *t*-test.

## 3 Results

### 3.1 Quantitative assessment of microglial morphology

In AD, microglia play dual and stage-dependent roles, facilitating amyloid and debris clearance during early disease but contributing to chronic neuroinflammation and synaptic dysfunction at later stages (Hansen et al., 2018; Zhang G. et al., 2021). Recent single-cell and spatial transcriptomic analyses have revealed that microglia acquire diverse activation states characterized by transcriptional modules related to metabolism and lipid handling, such as upregulation of ApoE, LPL, and TREM2, giving rise to discrete disease-associated microglial (DAM) and other neurodegeneration-linked activation clusters (Chen and Colonna, 2021; Loving and Bruce, 2020; Sun et al., 2023). Emerging evidence supports that modulating, rather than depleting, microglia offers a more effective strategy for restoring immune and homeostatic balance within the brain. Thus, microglia were selected for analysis as the principal immune effector cells of the central nervous system given their primary role as vigilant sentinels that continuously monitor the brain microenvironment and respond dynamically to injury or pathology. Because microglial morphology strongly mirrors activation state and function, our study focused on the quantitative assessment of structural parameters that can suggest activation phenotypes.

**FIGURE 1:**
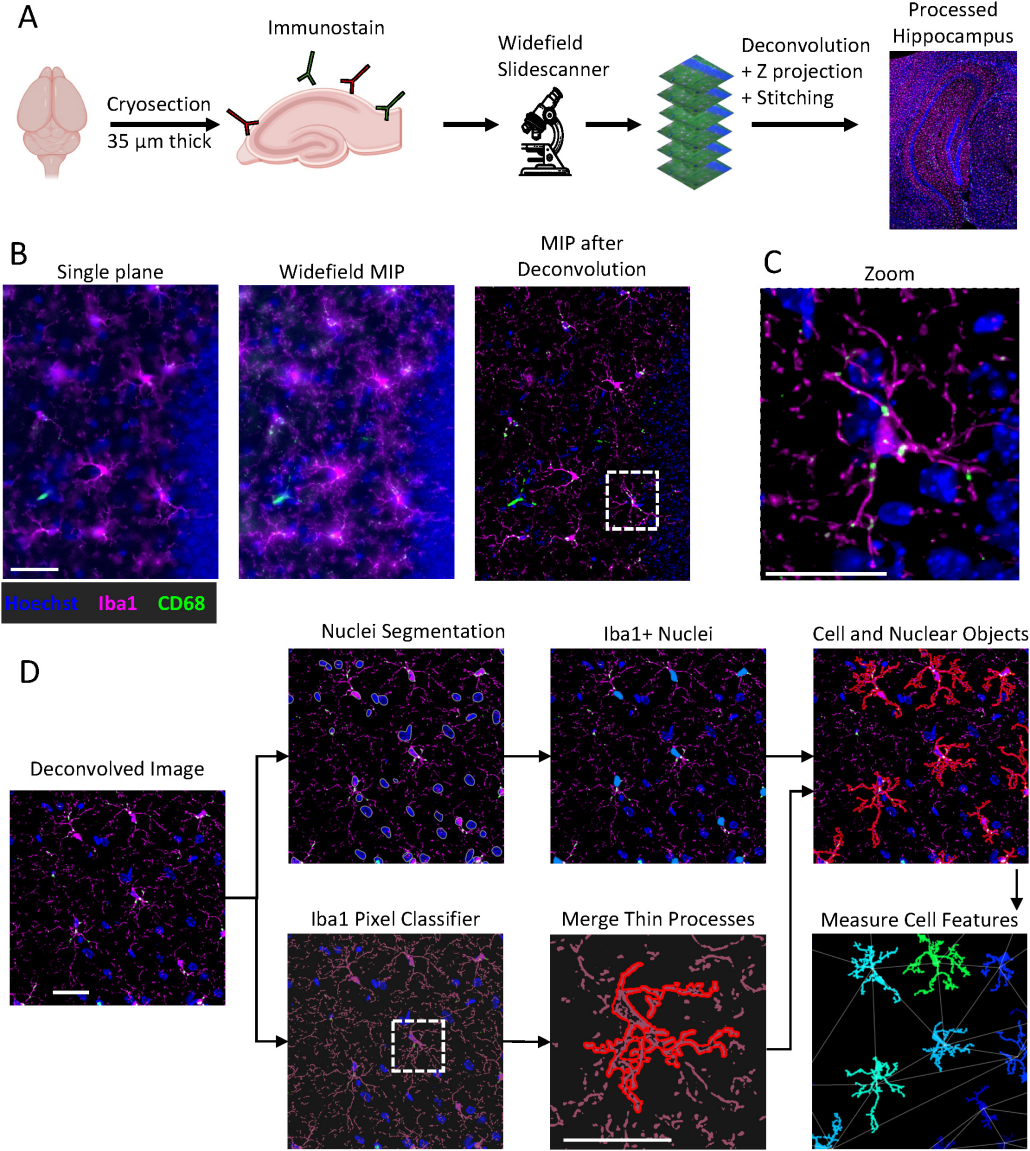
Workflow for assessing structural changes in microglial activation. **(A)** Free-floating 35-μm cryosections were immunostained for Iba1 and CD68 and imaged on a widefield slide scanner as Z-stacks (Δz = 0.49 μm). Tiles were deconvolved, maximum-intensity projected, and stitched to generate extended-depth, whole-hippocampus images. Scale bar = 50 μm. Icons come from bioicons.com and biorender.com. **(B)** Comparing single focal plane, maximum-intensity projection, or deconvolved maximum-intensity projection images shows that the deconvolution process yields higher spatial resolution and better signal-to-noise than the single plane or projected widefield images. Hoechst = blue, Iba1 = magenta, CD68 = green. Look-up tables are identical for the first two images, but adjusted for the deconvolved image to prevent oversaturation. Scale bar = 25 μm. **(C)** A zoom-in on the marked region of the deconvolved image shows crisp cell shape details and granular CD68 localization. Scale bar = 25 μm. **(D)** Microglia segmentation used a two-step pipeline: nuclei were segmented with Cellpose while a trained Iba1 pixel classifier delineated microglial arbors. Morphological filters merged thin processes and split touching objects. The cell outlines and nuclei were combined through the entire hippocampus and cell intensity features and shapes were measured, along with spatial relationships.

Briefly, 35 um sections from frozen mouse brains were cryosectioned and immunostained for CD68 and Iba1 using a free-floating method (Figure 1A). They were imaged with a widefield slidescanner, acquiring Z stacks with a 0.49 μm step size. The Z stacks were deconvolved and projected (Huygen’s Essential, Scientific Volume Imaging) to form a single image that captured a large depth of field. The resulting image contained more microglial processes than a single focal plane, but with improved clarity and signal-to-noise ratio compared to a maximum intensity projection of the entire stack (Figure 1B). CD68 expression is limited to discrete granules (Figure 1C). Segmentation and quantification of the microglia loosely followed the process in previous work (Leyh et al., 2021), but used the open-source analysis platform QuPath (Bankhead et al., 2017) for easier reproduction and adaptability. The code is available at Github. Microglia segmentation proceeded in 2 steps (Figure 1D). First, nuclei were segmented with Cellpose (Stringer et al., 2021) and filtered for Iba1 expression. Simultaneously, a pixel classifier was used to find all Iba1^+^ processes. Neighboring objects were merged through morphological closing, cells were filtered for those associated with an Iba1^+^ nuclei and overlapping cells were separated. The cell intensities, shape features, and spatial relationships were measured in QuPath and Fiji. A summary of microglial morphological and spatial parameters, and their association with microglial activation states, is shown in Supplementary Table 1.

### 3.2 Early hippocampal microglia remodeling in 5xFAD Mice: trending toward hyper-ramification in male 5XFAD microglia and increased density in females

**FIGURE 2:**
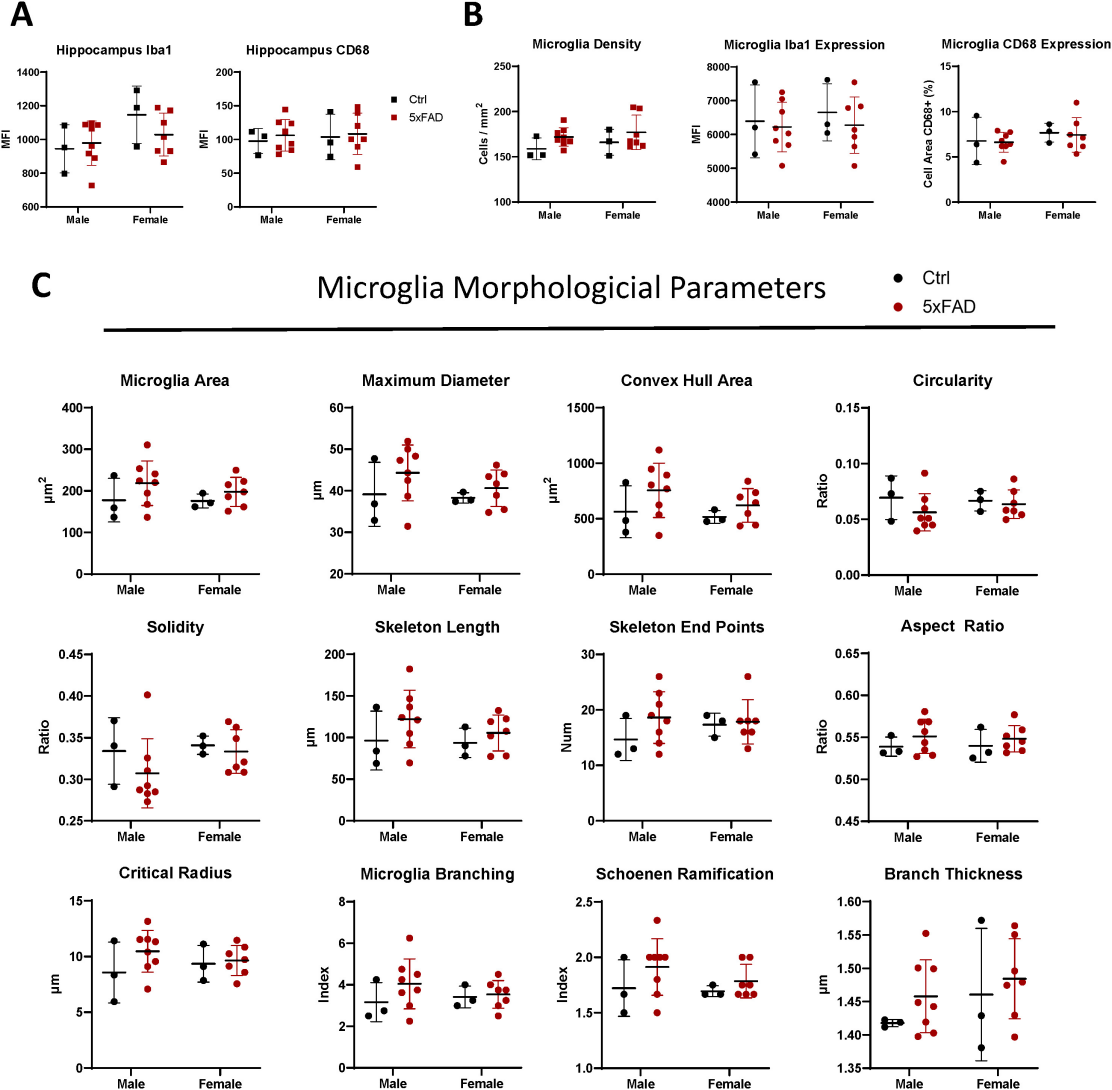
Early hippocampal microglia remodeling in 5xFAD Mice: trending toward hyper-ramification in male 5XFAD microglia and increased density in females. **(A)** Iba1 and CD68 immunoreactivity were quantified across the whole hippocampus to estimate total hippocampal microglial content and activation. Microglia were segmented and **(B)** the Iba1 Intensity and CD68 positive area, and **(C)** morphological features were measured per cell. Each square represents a measurement of 1 mouse; each circle represents the median value of all microglia in 1 mouse. Lines show mean ± standard deviation. Differences between genotypes and sexes were analyzed with 2-way ANOVA with a Fisher’s LSD *post-hoc* test — no comparisons were statistically significant.

The average hippocampus Iba1 and CD68 intensity gives an overall measure of microglial content. There were no significant differences between genotypes or sexes in either marker in the full hippocampal region, though the Ctrl females had slightly more Iba1 expression than the Ctrl males (Figure 2A). Focusing on segmented microglia, there were no statistically significant differences in average Iba1 or CD68 expression per microglia between sexes or genotypes (Figure 2B). However, the density of segmented microglia was slightly larger in the 5xFAD mice, though this is not seen in the Delaunay mean neighbor distance (Supplementary Figure 1A).

Looking beyond fluorescence intensity toward shape descriptors, we see no statistically significant differences between groups, but consistent trends toward hyper-ramification in the 5xFAD mice (Figure 2C). Microglia from 5xFAD brains are larger (higher cell area, diameter, convex hull area), more complex (lower circularity and solidity, higher skeleton length and number of skeleton end points) and more branched (higher aspect ratio, critical radius, branching index, and Schoenen Ramification Index), but with thicker branches, all features known to be correlated with hyper-ramified microglia (Heindl et al., 2018). Furthermore, the 5xFAD males, but not females, had a slightly larger distance between the microglia centroid and the nuclear centroid, suggesting asymmetric branch growth (Supplementary Figure 1B).

To understand how traditional microglia marker expression correlates with shape descriptors, the total population of microglia in the dataset (10,490 cells) were divided into deciles based on area, circularity, and branching index. The average CD68-positive area and average Iba1 intensity were calculated for the cells in each decile for each genotype. In both groups, CD68 and Iba1 expression correlate with decreasing area, increasing circularity, and decreasing branching index (Supplementary Figure 1C). Thus, microglial morphometrics trend with Iba1 and CD68 intensity, independent of sex and genotype.

### 3.3 Sex-specific changes observed in the CA1 hippocampal region.

Hippocampal atrophy is an early, robust imaging biomarker of AD that correlates with episodic memory decline and clinical progression (Fjell et al., 2014; Köhncke et al., 2021). Although oligomerization and fibrillization of Aβ most often begin in association neocortex, Aβ and tau pathology subsequently involve medial temporal circuits, with early tau spread across entorhinal to hippocampal subfields including CA1 (Escamilla et al., 2024; Xu et al., 2021). CA1 pyramidal neurons constitute a principal hippocampal output stage within the trisynaptic circuit, supporting temporal coding, sequence memory, and spatial navigation along with high metabolic demand and dense glutamatergic/NMDA signaling, contributing to selective CA1 vulnerability (Alfaro-Ruiz et al., 2024; Shipton et al., 2022). Human and animal studies consistently show early CA1 synaptic/neuronal loss and neurofibrillary tangle accumulation that track with memory impairment (Zhang G. et al., 2021). Microglia are key effectors of this vulnerability in CA1. In 3xTg-AD and other models, microglial density and activation rise in CA1 around the time plaques and tangles emerge, with CA1 showing stronger glial reactivity and neurodegeneration than neighboring subfields (Nairuz et al., 2024; Rodríguez et al., 2010; Rodríguez et al., 2013; Ugolini et al., 2018). Mechanistically, complement-tagged synapses in the hippocampus are aberrantly engulfed by microglia early in disease, providing a causal route from microglial activation to CA1 synapse loss and cognitive decline (Carpanini et al., 2022; Hong et al., 2016; Shi et al., 2017; Wu et al., 2019). These studies show that the CA1 region is central to hippocampal computation, displays early and selective degeneration in AD, and that microglia-mediated synaptic pathology is tightly linked to memory loss. Given that the CA1 region is among the earliest and most vulnerable sites of neurodegeneration in AD (Ginsberg et al., 2010; Ugolini et al., 2018), we sought to determine whether microglia within the CA1 in these young mice display greater structural changes between genotypes and sexes than could be found when averaging the entire hippocampus region.

**FIGURE 3:**
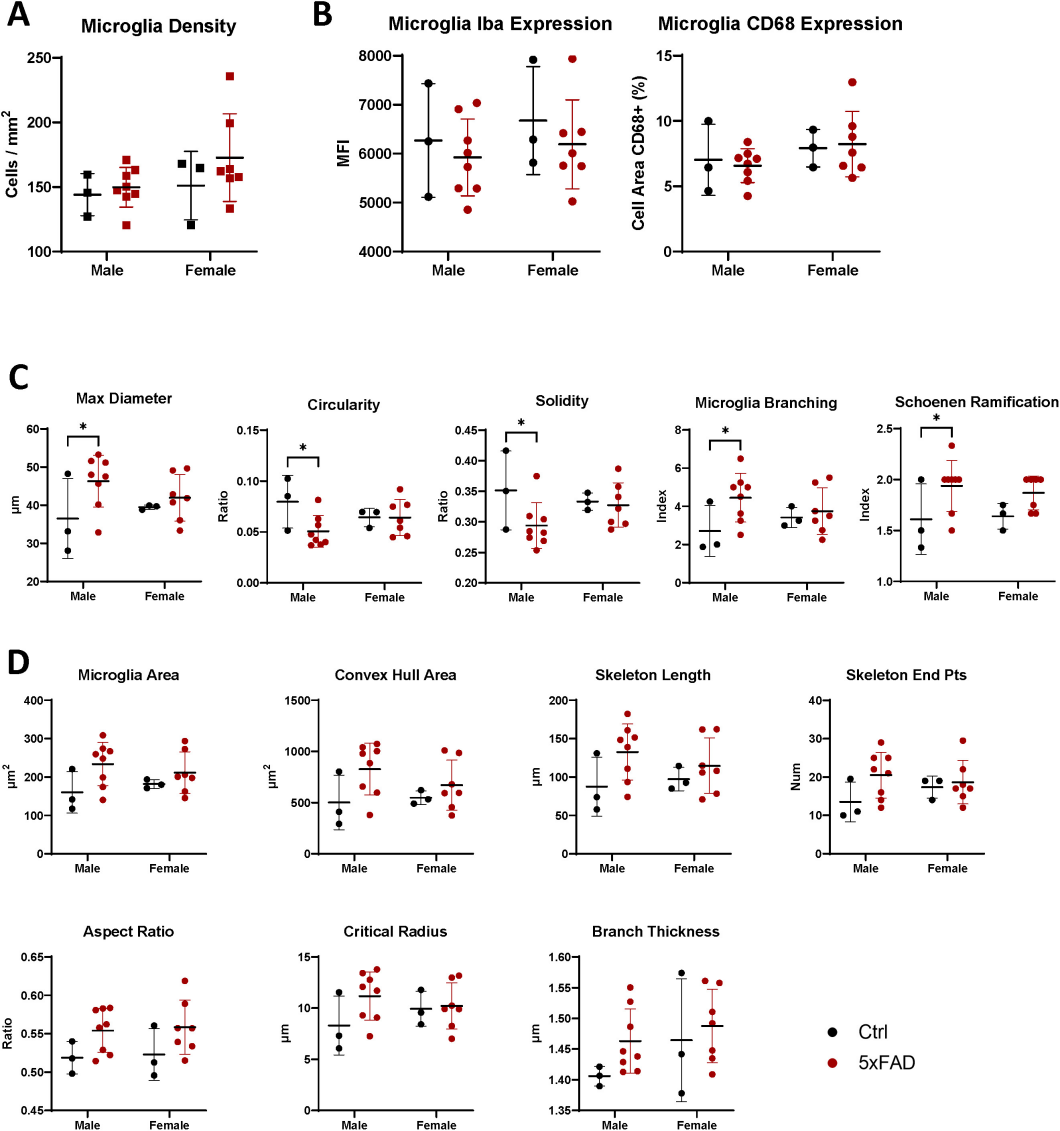
Sex-specific changes observed in the CA1 hippocampal region. **(A)** The overall density of microglia in the hippocampal CA1 region of male and female, 5xFAD and Ctrl mice. **(B)** Microglial expression of Iba1 and CD68 in the CA1 region show no changes between sex or genotype. **(C)** Microglia from the CA1 region of male 5xFAD mice show significant changes in solidity, circularity, and branching index compared to Ctrl mice. **(D)** Other morphological parameters show a trend toward 5xFAD microglia being more hyper ramified than controls, with a larger difference in males. Each square represents a measurement of 1 mouse; each circle represents the median value of all microglia in 1 mouse. Lines show mean ± standard deviation. *p < 0.05, **p < 0.01 by 2-way ANOVA with a Fisher’s LSD *post-hoc* test.

Within the CA1 region, there were no significant changes in microglial density, Delaunay distance, or cellular Iba1 or CD68 expression (Figures 3A, B and Supplementary Figure 1D). However, looking at morphological changes in the microglia in the CA1 region, we see many of the trends for more complex shapes in the 5xFAD mice are more pronounced than in the full hippocampus, particularly in male mice. Male 5xFAD microglia had significantly lower circularity and solidity metrics and higher maximum Feret diameter, branching index, and SRI when compared to the male Ctrl microglia (*p* < 0.05 for all, Figure 3C). Furthermore, many of the parameters associated with hyper ramification of 5xFAD mice are more pronounced in the CA1 region than in the full hippocampus, including cell size (17.8% vs. 30.3% increase in 5xFAD compared to control in full hippocampus and CA1-only, respectively), hull area (27.4% vs. 42.5%), skeleton length (19.9% vs. 33.8%), skeleton end points (14.0% vs. 26.9%), aspect ratio (1.9% vs. 6.8%), critical radius (12.2% vs. 17.3%), and centroid offset (0.4% vs. 3.3%) (Figure 3D and Supplementary Figure 1E). In these cases, the males have the largest differences between genotypes.

### 3.4 Defining early reactive microglia in the female 5xFAD mice

**FIGURE 4:**
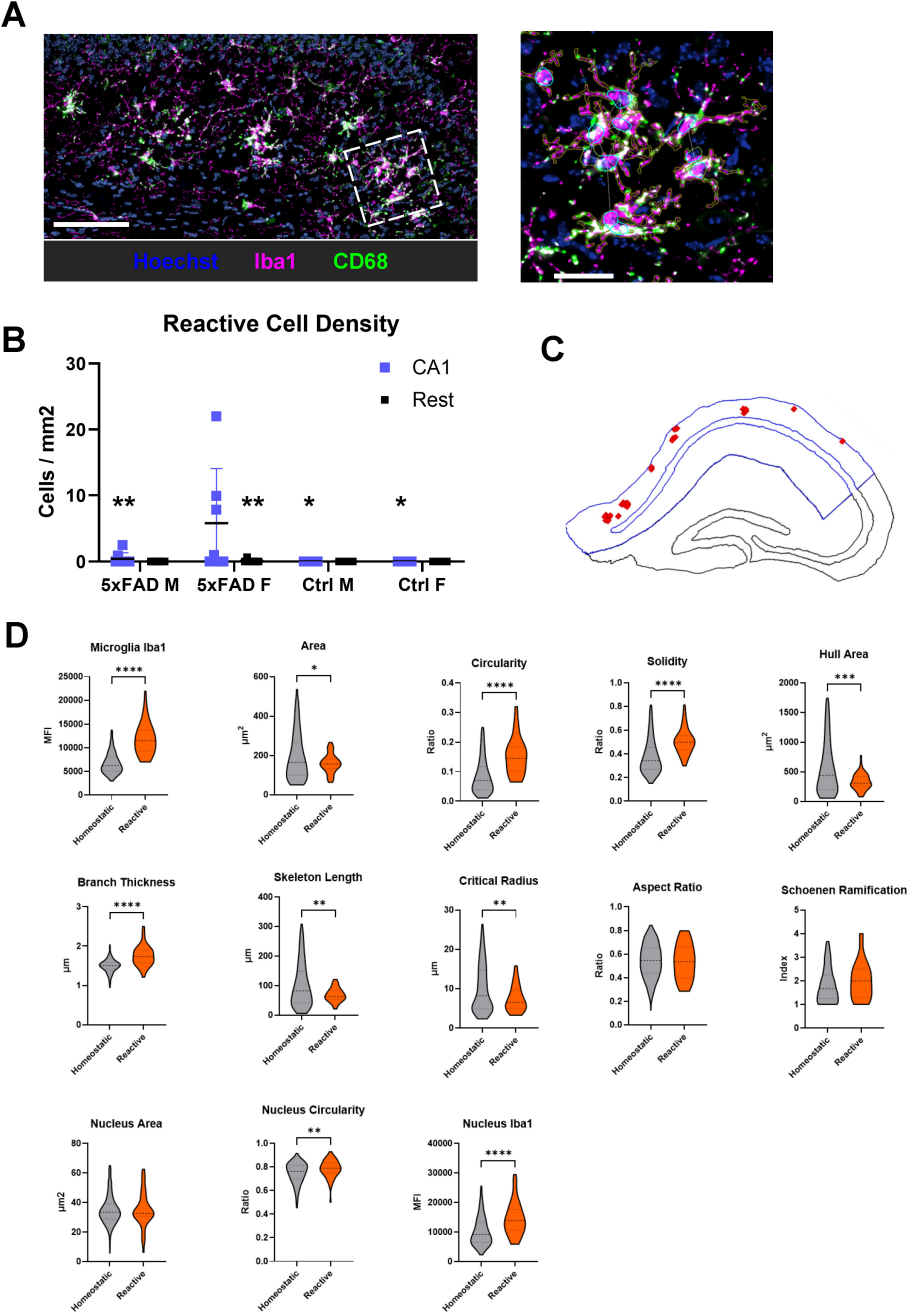
Defining early reactive microglia in the female 5xFAD mice. **(A)** (Left) An image from the CA1 region of a female 5xFAD mouse showing reactive microglia- with visibly high CD68, thick branches, and clustered nuclei. Scale bar = 50 μm. (Right) A zoom-in on the marked region, with the cell and nuclear segmentation, and nuclear connections visible (brown, cyan, and white, respectively). Scale bar = 25 μm. Hoechst = blue, Iba1 = magenta, CD68 = green. **(B)** The density of all reactive microglia, separated by location, genotype, and sex. Asterisks show significant differences compared to the female, 5xFAD CA1 region. * = *p* < 0.05, ** = *p* < 0.01 by 2-way ANOVA with a Fisher’s LSD *post-hoc* test. 3 of the mice have substantially more reactive microglia than the others. **(C)** Location of the reactive cells (red squares) in a representative female 5xFAD hippocampus. The size of the squares has been enlarged for visualization. **(D)** Comparing the morphological and intensity features of the 50 reactive cells found in these mice against the remaining microglia in these 3 mice (CA1 region only), we can see that these cells have changes typical of reactive microglia compared to homeostatic: thicker branches, smaller size, more circular shape, and higher Iba1 expression. ***p < 0.001, ****p < 0.0001 using an unpaired *t*-test after ROUT outlier removal with a 1% threshold. Dotted lines show median and quartiles.

The emergence of highly reactive, amoeboid microglia, typically localized around amyloid plaques during later stages of pathology in AD mouse models is one of the hallmarks of disease progression. Studies demonstrate that by 2–4 months of age, coinciding with the initial stages of plaque formation, microglia in 5xFAD mice adopt an amoeboid morphology characterized by retracted, thickened processes and increased immunoreactivity for activation markers such as Iba1 and F4/80, consistent with a reactive state (Boza-Serrano et al., 2018; Gyoneva et al., 2016; Hong et al., 2016; Hou et al., 2022; Yuan et al., 2016) CD68 is associated with microglia activation and inflammatory states (Jurga et al., 2020). Furthermore, microglial transcriptome changes in 5xFAD mice by 4–6 months indicate the upregulation of inflammatory and immune genes, complement and integrin signaling, and genes linked to phagocytosis. This is consistent with reactive/disease-associated microglia phenotypes (Boza-Serrano et al., 2018; Gyoneva et al., 2016; Hou et al., 2022; Yuan et al., 2016). In our current study of 6–8 week old mice, we noticed infrequent cells that have high CD68 expression (≥ 35% positive), at least medium branching (branching index ≥ 1), and that are clustered together (nucleus centroid <25 μm from another nucleus centroid) that appear to be active/reactive microglia (Figure 4A). The female 5xFAD mice showed a greater incidence rate of reactive microglia in the CA1 region than in the rest of the hippocampus or compared to male 5xFAD mice or Ctrl mice (Figure 4B, Supplementary Figure 1F). These cells were almost exclusively found in the outer section of the CA1 region (Figure 4C). In the 3 female 5xFAD mice with a substantial frequency of these reactive cells, we found 50 cells across the CA1 regions. Comparing the feature profile of this subset of microglia against the remaining microglia in these 3 mice (CA1 region only), we can see that these cells have the typical reactive microglia profile: higher Iba1 expression, slightly contracted with smaller area, hull area, skeleton length, and critical radius but higher circularity, solidity, and branch thickness (Figure 4D). Thus, although we did not find classically amoeboid microglia in 5xFAD mice at this stage in AD development, we did detect a shift toward early reactivity, specifically in the female 5xFAD CA1 region. These findings support our hypothesis (grounded in prior reports of earlier and stronger microglial activation in female AD models) that female microglia transition to reactive phenotypes sooner than males.

## 4 Discussion

In this study, we provide evidence that microglial remodeling at the pre-plaque stage of 5xFAD mice is both measurable and sex-dependent, underscoring the value of our open-source morphometric pipeline. We observed that male 5xFAD mice exhibited hyper-ramified microglia, whereas female 5xFAD mice displayed a more reactive phenotype, particularly in the CA1 region. In the AD brain, microglia typically transition from a ramified surveillance morphology (high circularity and solidity, modest branching) toward an activated phenotype characterized by enlarged soma, retracted processes, reduced arborization and increased amoeboid shape. Thus, our finding of lower circularity/solidity and higher branching index suggests that these microglia are in a morphometric state distinct from classical amoeboid activation. One plausible interpretation is that we are capturing an early “hyper-ramified” or “primed” microglial phenotype characterized by increased branching and process length in the face of evolving amyloid pathology rather than a fully collapsed amoeboid state. While literature in human AD tissue reports fewer highly ramified microglia near plaques [increased reactive and amoeboid (Davies et al., 2017; Franco-Bocanegra et al., 2021)], our data may reflect an earlier phase of microglial morphological adaptation in the 5xFAD model. Consistent with prior microglial morphometric work, reduced circularity or solidity together with increased branching is compatible with a more complex, hyper-ramified or primed microglial state rather than a classic amoeboid phenotype (Fernández-Arjona et al., 2017; Reddaway et al., 2023; Zanier et al., 2015). These findings align with prior literature demonstrating that microglial changes emerge well before overt amyloid plaque deposition. Boza-Serrano et al. reported that inflammatory alterations are detectable in microglia of 5xFAD mice as early as 6 weeks, preceding amyloid accumulation (Boza-Serrano et al., 2018). Similarly, Chithanathan et al. documented early microglial activation and morphological changes in two-month-old male 5xFAD mice (Chithanathan et al., 2022). Our finding of hyper-ramification in males may therefore represent an exaggerated surveillance response to a subtly changing neuroenvironment, consistent with a primed yet not fully reactive microglial state. In contrast, female 5xFAD microglia more closely resemble disease-associated microglia. Disease-associated microglia have been characterized by increased glycolytic metabolism and antigen presentation, while having reduced phagocytic activity (Guillot-Sestier et al., 2021). This sex-biased divergence is consistent with clinical and preclinical observations that females often demonstrate heightened neuroinflammation and accelerated amyloid pathology (Casaletto et al., 2022). By capturing these structural differences with quantitative parameters, our tool allows us to dissect early microglial transitions in a manner that is reproducible and scalable across datasets. Importantly, correlations between microglial structural complexity and canonical activation markers, including Iba1 and CD68, are maintained irrespective of sex or genotype (Supplementary Figure 1C). This conserved association underscores a fundamental coupling between cytoskeletal remodeling and phagolysosomal activity, reinforcing morphological features as quantifiable indicators of microglial functional state. Collectively, these data establish morphometric profiling as a sensitive and unbiased approach to detect early microglial activation, while capturing sex-specific trajectories before amyloid deposition.

In the present study, we focused on developing an image analysis method to semi-automatically quantify microglial parameters and activation states in murine hippocampuses. We characterized >10,000 microglia in 21 mice, making this among the largest studies of microglial shape to date (Reddaway et al., 2023). Performing the analysis on the entire hippocampus, instead of individual ROIs, allowed us to find rare reactive cells in young mice. Using a pixel classifier to detect Iba along with morphological processing to merge small branches enables more accurate segmentation than thresholding alone. Additionally, incorporating the Hoechst channel to segment nuclei made it easier to separate clustered cells and therefore identify the rare, reactive microglia. By making the code available open-source, we hope to provide a useful tool to the community. While future users will have to train their own pixel classifier on their data, further steps for cell segmentation and morphometric analysis should be automated.

Due to the low number of reactive cells present in the dataset, we could not rely on a supervised or unsupervised classifier to identify cell phenotypes, as was done in previous work (Heindl et al., 2018; Leyh et al., 2021). Instead, we used simple thresholds on only a few parameters that can easily be repeated on smaller datasets. Frequently, microglia analysis is performed on single focal planes (getting only a fraction of each cell) (Heindl et al., 2018; Leyh et al., 2021), widefield Z projections (which blurs fine details), or confocal Z stacks (low-throughput) (Heindl et al., 2018; Leyh et al., 2021). By acquiring a large Z range, and using deconvolution to reduce the effects of out-of-focus light, we were able to get more of the microglia branching at the speed of widefield imaging. While the software we used for deconvolution in this work is commercial, similar processing can be implemented in open-source software (Sage et al., 2017). Here, we also introduce two uncommon metrics for microglia: centroid offset (the distance between the cell centroid and nucleus centroid) and Delaunay mean distance (a measure of cell density). We note that microglia in the CA1 region of 5xFAD mice show higher centroid offset while also trending toward hyper ramification. Similarly, reactive cells in the female 5xFAD mice tend to be more clustered. Future work will be needed to determine if this pattern is coincidental or consistent.

Given that morphological changes contribute to the function and activity of microglia, regional differences in microglial branching enable these cells to tailor their surveillance, synaptic maintenance, and immune functions to the specific physiological and metabolic demands of each brain region. Therefore, under homeostatic conditions, microglia in different regions exhibit distinct morphologies. Hippocampal microglia exhibit higher turnover and a more reactive baseline phenotype than cortical microglia, and in some studies appear slightly less ramified, reflecting adaptation to the high synaptic and metabolic activity of the hippocampal environment (Tan et al., 2020; Tay et al., 2017). Basal ganglia microglia show region specific branching nucleus accumbens and SNr microglia are highly branched, whereas VTA and SNc microglia are more sparsely ramified (De Biase et al., 2017). These baseline differences suggest that local cytoarchitecture influences microglial arborization. In AD pathology, region-specific microglial changes have also been reported. In Alzheimer’s disease, human studies report a reduction in the population of highly ramified “surveying” microglia (fewer cells with long, thin highly branched processes) in the hippocampus and associated cortical regions compared to controls, aligning with increased Aβ load and morphological simplification of microglia. In human AD, multiple studies report a shift away from highly ramified surveying morphologies toward less-ramified or reactive states, including quantitative reductions in total process length and branch number in temporal cortex, and a reduced fraction of ramified microglia overall (Franco-Bocanegra et al., 2021; Singh-Bains et al., 2019). While morphometric comparisons across prefrontal, entorhinal, and hippocampal subfields remain limited, the convergent evidence supports region-dependent baseline states and AD-associated loss of complex branching in vulnerable medial temporal lobe structures. These studies cumulatively suggest thar microglia in these regions assume activated morphologies sooner, correlating with local pathology. Our imaging pipeline, which quantifies microglial arbors, enables direct comparisons across brain regions. This is valuable for detecting such heterogeneity in microglial structure and reactivity between regions like prefrontal cortex, entorhinal cortex, and hippocampus, providing deeper insight into region-dependent microglial responses in AD.

This study has several limitations. First, the number of control animals analyzed was limited (*n* = 3), which may contribute to variability and reduced statistical power. Nonetheless, all controls were age- and sex-matched non-transgenic littermates processed in parallel with 5xFAD mice to minimize technical confounders. Second, all 5xFAD mice used were hemizygous and obtained directly from the vendor, and parental origin of the transgene (maternal versus paternal inheritance) was not controlled. Recent work has shown that paternal inheritance can influence amyloid pathology (Sasmita et al., 2025), which may underlie some of the intra-group variability observed. Finally, the study was designed primarily to establish and validate a reproducible workflow for quantifying microglial morphology rather than to draw definitive biological conclusions. Future studies with larger cohorts, controlled breeding strategies, and functional readouts will be essential to strengthen mechanistic interpretations and link morphological diversity to microglial activation states. Although we did not directly investigate lipid alterations, this analytical framework provides a foundation for future studies aimed at integrating peripheral bioactive lipid profiles with microglial remodeling in murine AD models. Prior work has demonstrated that microglia are key contributors to lipid-mediator signaling: for example, microglial production of leukotrienes has been shown to exacerbate neuroinflammation in APP/PS1 mice, and genetic or pharmacological inhibition of 5-Lipoxygenase and its activating protein reduces pathology and improves outcomes (Michael et al., 2020). Conversely, specialized pro-resolving mediators derived from polyunsaturated fatty acids are diminished in AD and, when supplemented, can restore microglial phagocytosis and synaptic function (Hou et al., 2022; Miyazawa et al., 2020). These findings highlight the importance of lipid-microglia interactions as both biomarkers and therapeutic entry points. By applying our morphometric tool in conjunction with serum lipidomics, we aim to identify correlations between distinct microglial activation states and circulating lipid mediators. Such integration has the potential to define early molecular signatures of disease, inform patient stratification, and uncover druggable pathways that shift microglia from detrimental to homeostatic states. In this way, our current work not only advances methodology for microglial characterization but also lays the groundwork for mechanistic and translational studies linking neuroinflammation, lipid metabolism, and therapeutic targeting in AD.

## Data Availability

The datasets presented in this study and the image analysis scripts can be found in online repositories. The names of the repository/repositories and accession number(s) can be found below: https://www.ebi.ac.uk/biostudies/bioimages/studies/S-BIAD2332, https://github.com/saramcardle/Image-Analysis-Scripts/tree/master/Microglia%20Analysis%20in%20QuPath

## Footnotes

1
https://github.com/saramcardle/Image-Analysis-Scripts/tree/master/Microglia%20Analysis%20in%20QuPath

## Supplementary material

Supplementary Figure 1 **(A)** Delaunay clustering was performed on all microglia in the hippocampus based on the cell centroids. For each cell, the mean distance to its neighbors was calculated in QuPath. Each dot shows the median value of all cells in 1 mouse. **(B)** The distance between the cell centroid and the nucleus centroid was also calculated for each cell. **(C)** By dividing the microglia into deciles based on area, circularity, or branching index, we can see clear correlations in these morphological parameters with CD68 and Iba1 expression. Lines show SEM per data point. **(D,E)** Delaunay clustering and nuclear centroid offset was calculated on the cells in the CA1 region alone. In A, B, D, and E, differences between genotypes and sexes were analyzed with 2-way ANOVA with a Fisher’s LSD *post-hoc* test — no comparisons were statistically significant. Lines show mean ± standard deviation. **(F)** (Left) An image from the CA1 region of a female Ctrl mouse, taken from approximately the same anatomic region as Figure 4A, showing standard ramified cells. Scale bar = 50 μm. (Right) A zoom-in on the marked region, with the cell and nuclear segmentation visible (brown and cyan, respectively). Scale bar = 25 μm. Hoechst = blue, Iba1 = magenta, CD68 = green.

Supplementary Table 1 Summary of parameters used to assess microglial morphology, complexity, and branching intensity. Cell area, diameter, and convex hull area constitute microglial size parameters. Circularity, solidity, skeleton length, and number of skeleton end points constitute complexity parameters. Aspect ratio, critical radius, branching index, and SRI constitute branching parameters.
