# Supplementary figures and images for: Unmasking early microglial remodeling in an Alzheimer’s disease mouse model
Source: Front Cell Neurosci. 2026 Jan 2;19:1720382. doi: 10.3389/fncel.2025.1720382 (PMC12807895; doi:10.3389/fncel.2025.1720382)

# Supplemental Figure 1

Full Hippocampus

CA1 Region Only

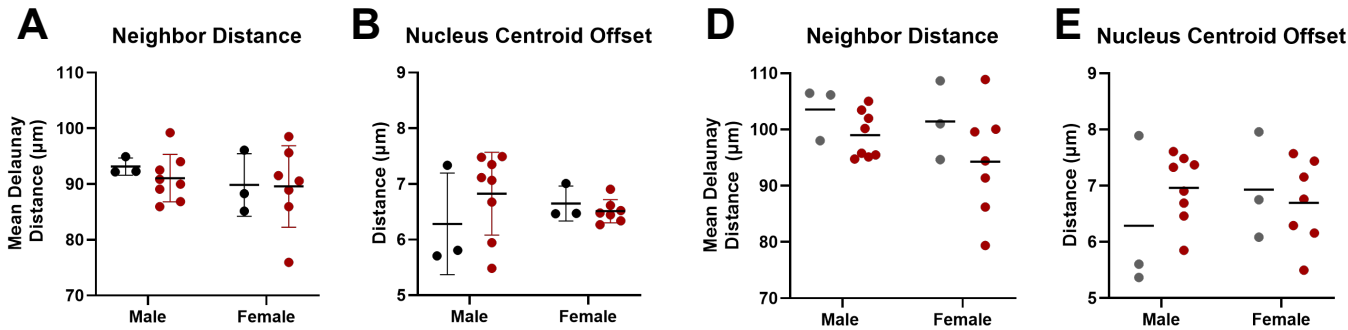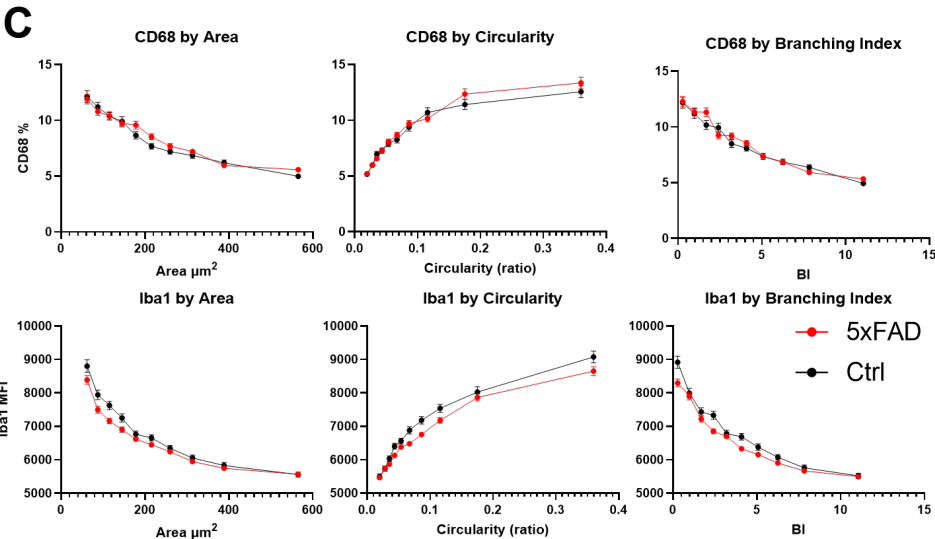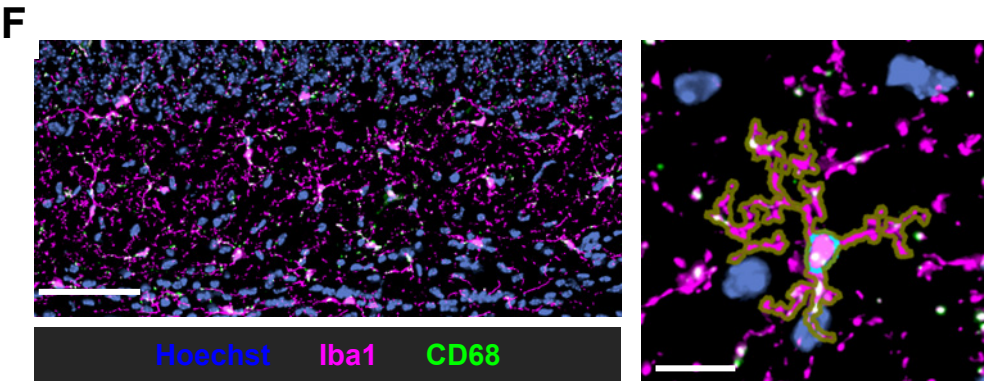

Supplement: Supplementary Figure 1 — (A) Delaunay clustering was performed on all microglia in the hippocampus based on the cell centroids. For each cell, the mean distance to its neighbors was calculated in QuPath. Each dot shows the median value of all cells in 1 mouse. (B) The distance between the cell centroid and the nucleus centroid was also calculated for each cell. (C) By dividing the microglia into deciles based on area, circularity, or branching index, we can see clear correlations in these morphological parameters with CD68 and Iba1 expression. Lines show SEM per data point. (D,E) Delaunay clustering and nuclear centroid offset was calculated on the cells in the CA1 region alone. In A, B, D, and E, differences between genotypes and sexes were analyzed with 2-way ANOVA with a Fisher’s LSD post-hoc test — no comparisons were statistically significant. Lines show mean ± standard deviation. (F) (Left) An image from the CA1 region of a female Ctrl mouse, taken from approximately the same anatomic region as Figure 4A, showing standard ramified cells. Scale bar = 50 μm. (Right) A zoom-in on the marked region, with the cell and nuclear segmentation visible (brown and cyan, respectively). Scale bar = 25 μm. Hoechst = blue, Iba1 = magenta, CD68 = green. [file Data_Sheet_1.pdf]
